# Indigenous knowledge approach in maintaining a livelihood in the face of disastrous climate change: Case of drought in Msinga villages, KwaZulu-Natal

**DOI:** 10.4102/jamba.v11i1.758

**Published:** 2019-11-14

**Authors:** Joseph R. Rukema, Beatrice S. Umubyeyi

**Affiliations:** 1 School of Social Sciences, College of Humanities, University of KwaZulu-Natal, Durban, South Africa; 2 School of Built Environment and Development Studies, College of Humanities, University of KwaZulu-Natal, Durban, South Africa

**Keywords:** indigenous, approach, livelihood, disastrous, climate change, Msinga, KwaZulu-Natal province, South Africa

## Abstract

The aim of this study is to explore and examine the ways of maintaining livelihoods of communities in the face of extreme climatic conditions using indigenous knowledge systems. Special attention was paid to drought prevailing in the Msinga village communities in the northern part of KwaZulu-Natal. This is a qualitative study. In order to achieve its objectives, this study used semi-structured interviews. In total, there were 16 participants from the Msinga villages. The findings demonstrated that droughts are endemic to the study area, and drought-management strategies are as intrinsic to local livelihood systems as are seasonal-adjustment strategies. The findings also indicated that communities in Msinga have knowledge of drought management. However, this knowledge contributes very little to the management of drought. Limitations in traditional knowledge that contribute effectively to drought management are due to a number of factors including conflicts between traditional knowledge and modern science. This implied that most young people are no longer interested in traditional knowledge but are more interested in modern science. Furthermore, government programmes and interventions hamper the efforts of traditional knowledge in dealing with the consequences of drought and thereby make the community vulnerable to drought.

## Introduction

The Msinga local municipality is located in the northern part of KwaZulu-Natal. Msinga is predominantly mountainous, with rolling hills, loose stones and rocks, which make it difficult for farming. According to the Institute of Natural Resources (2007), only 40% of the land has the potential for farming, but subsistence farming remains the major economic activity in the area. Added to this is the limited capacity of the land for productive agricultural development due to poor soil quality, adverse climatic conditions and poor agricultural practices, such as overgrazing (Msinga Local Municipality 2004 – Integrated Development Plan 2004/2005). Despite the large irrigation potential covering up to 40% of agriculture practice, the area is subjected to water shortages, high soil erosion and low land carrying capacity (Institute of Natural Resources 2007).

A study conducted by the Institute of Natural Resources in 2007 reveals that Msinga is characterised by an annual rainfall of between 600 mm and 700 mm. In terms of international standards, this indicates drought of a high magnitude (Institute of Natural Resources 2007). The South African Weather Services (KwaZulu-Natal Station, pers. comm., June 2008) demonstrates that the northern part of KwaZulu-Natal, which includes Msinga, is prone to dry conditions. The changing nature of rainfall in most areas of Msinga makes it risky to invest in the production of crops, such as maize, vegetables and sorghum, which are the main sources of food in these areas. Msinga suffers intermittent and periodic droughts, with the last officially recorded drought occurring in 2004 (Drought Information Bulletin 2004). However, despite the drought, the findings of this study showed that the communities in Msinga, in particular, continue to experience the effects of drought after this period, and its affects are severe (Personal interviews with members of Msinga). The researcher at the Maurice Webb Race Relations Unit (MWRRU 2006) supported the above argument by reporting that Msinga has high levels of illiteracy, reaching up to 79%. In his study he adds that 95% of households in the area rely on wood as fuel for cooking which places a burden on the already vulnerable environment. Drought conditions in Msinga are aggravated by the prevailing economic and social situation in the area. Therefore, there is no doubt that droughts have caused complex socio-economic consequences for members of Msinga communities and trigger vulnerability in the livelihood of these communities.

The aim of this study is to investigate the traditional or indigenous knowledge of members of a rural community in Msinga in relation to drought. Mwaura (2008:21) defines traditional and indigenous knowledge as ‘specific systems of knowledge and practice, developed and accumulated over generations within a particular cultural group and region, and as such are unique to that group and region’. The International Institute for Rural Reconstruction (IIRR) (1996:7) considers indigenous knowledge as that knowledge ‘that people in a given community have developed over time, and continue to develop, based on experience, often tested over centuries of use, adapted to local culture and environment, which are dynamic and changing’. Analysing traditional knowledge in this context, enables it to be understood as a tool and a combination of skills that help communities or households to withstand uncertainty, and adjust to changes in living conditions (Mwaura 2008). Potteir, Bicker and Sillitoe (2003:21) are convinced that traditional and indigenous knowledge play a central role in developing the self-reliance of a society or community, and have been recognised as important by many disciplines in the development sector. In relation to communities’ indigenous disaster management strategies, Save the Children UK (2000) clearly understands traditional knowledge as one that needs to take into account the daily survival strategies of the communities, as this enables one to make sense of the complex ways in which individuals, households and communities achieve and sustain their livelihoods, as well as the likely impact of an external shock on such communities. Thus, understanding traditional and indigenous knowledge fits into the broader reconstruction and development of strategies in South Africa. It provides a new platform for self-discovery. Problems are identified, and solutions are built on knowledge of grassroots communities (Okumu 2002).

Several global imperatives, such as the World Health Organization (WHO), the United Nations Environmental Programme (UNEP) and the United Nations Development programme (UNDP), also underpin the need for renewed attention to the communities’ knowledge of managing drought effectively (Hoppers 2002:257). Some communities have a vast pool of knowledge on prediction and early warning signs that help them prepare and manage drought (Hoppers 2002:257). For instance, the Luo community in Kenya had a large number of climate monitoring indicators that enabled them to tell when it was the right time to start planting in anticipation of the rains or to preserve and store food in anticipation of a dry season. These indicators include observation of the behaviour of animals, birds, reptiles, amphibians, insects, vegetation and trees, winds, temperatures and celestial bodies (Mwaura 2008:22).

In line with climatic uncertainties, communities have also devised a variety of measures such as growing drought-resistant crops. The Luo community shifted from maize to cassava and early-maturing indigenous crop varieties, wetlands cultivation and livestock diversification. These strategies have enabled them to survive climatic hazards with little or no support from the outside world (Mwaura 2008:21). In other words, over the years, as a particular group deals with its survival, it develops its own practical ways to deal with problematic situations.

It is for these reasons this study attempts to explore and examine the strategies of the communities of the Msinga villages in maintaining their livelihood in the face of extreme climatic conditions with a particular focus on drought.

## Livelihood, traditional knowledge and drought management

This section provides a comprehensive examination of common households’ indigenous responses to drought and the maintenance of livelihood. Local traditional knowledge responses to disasters revealed that there are a number of mechanisms, which communities or households adopt to predict, mitigate and help them to cope during drought periods (Altieri & Koohafkan 2008). Rahmato (1991), examining communities’ traditional knowledge responses to drought in Ethiopia, argues that household knowledge to drought management techniques may be either crude or elaborate. They depend on a number of variables, such as the frequency of droughts experienced, available resources at hand and the social, cultural and political situation in the affected communities (Rahmato 1991:137).

Rahmato (1991) adds that what influences the households’ response is associated with the households’ livelihoods and everyday activities, as the latter serve as the determining factors of their ability to adjust to the changes brought about by drought and external shocks. Examining communities’ responses to drought in South Africa, Mwaura (2008) states that the households indigenous response strategies to drought are mainly influenced by culture and belief systems and the economic situation.

The following section provides an in-depth examination of households’ indigenous response strategies to drought and how these help households maintain livelihoods in the face of shocks or uncertainty.

The concept ‘preparedness’ is defined by the United Nations International Strategy for Disaster Reduction (2002) as:

Activities and measures taken in advance to ensure effective response to the impact of disasters, including the issuance of timely and effective early warnings and the temporary removal of people and property from a threatened location. (p. 25)

In response to drought, households have developed a vast storehouse of knowledge on prediction and early warning signs that help them to prepare and manage drought effectively, even sometimes relocating whenever possible. As discussed earlier, there are diverse ways that individuals or households are influenced to adopt particular response strategies. In the field of prediction and early warning of disasters, communities in Kenya have a large number of climate monitoring indicators that enable them to know when it is the right time to start planting in anticipation of the rains or to preserve and store food in anticipation of a dry season (Mwaura 2008:21). These indicators include observation of the behaviour of animals, birds, reptiles, amphibians, insects, vegetation and trees, winds, temperatures and celestial bodies, such as the size of the moon and stars (Mwaura 2008:21). When a drought is predicted, the community devises strategies that may help them weather the shocks brought about by these disasters. Amongst the most common strategies are building stores and savings which could assist as a fallback (Chen 1991; Mwaura et al. 2008).

Many communities may face similar hazards, but the measures adopted will differ. Mwaura (2008) examined households’ responses to disaster and drought in four African countries: Kenya, Tanzania, Swaziland and South Africa. There are similarities and differences in the households’ early warning and preparedness system. This study conducted by Mwaura (2008) shows that in Kenya, the presence of ‘snakes and other reptiles, as well as wild animals, around homesteads in search of water and food’, in many areas indicated the prevalence and continuation of a drought (Mwaura 2008:65).

As Mwaura (2008) states, in South Africa, the common belief is that drought is:

[*A*]n act of God and therefore the early warning indicators have to be revealed to elders by ancestors who also act as intermediaries between nature and God to prevent drought from happening. (p. 66)

As Mwaura (2008) points out, in Swaziland, indigenous methods used to predict drought included:

[*A*]bundance of butterflies during the farming season, presence of army worms, the dropping off of young avocado fruits, and the abundance of wild fruits during the months of December to February. (p. 67)

The findings from this study revealed a number of traditional methods used by specialised community elders to predict drought and famine. These included ‘reading signs on goat intestines, social conflicts, diseases, childbirth, peace or war in the chiefdom, and so forth’. As Mwaura (2008) points out:

If the small intestine was found to be empty, drought or famine or hostility and war were to be expected in the chiefdom but if it had a lot of dung this foretold plenty of rain, no famine and peace. (p. 69)

## Drought mitigation and prevention

The United Nations International Strategy for Disaster Reduction (2002:25) defines the concept ‘mitigation’ as ‘structural and non-structural measures undertaken to limit the adverse impact of natural hazards, environmental degradation and technological hazards’.

Studies demonstrate that most households attempt to integrate flexibility into their basic livelihood systems in order to minimise external shocks (Chen 1991; Mwaura 2008). Flexibility is most often directed at production systems, such as diversification of crops and livestock, in the use of different resources, deployment or recruitment of labour and in the mix of different occupations and activities, such as seeking employment (Chen 1991:110). Cultivators mix crops and animal husbandry to varying degrees, mix and rotate crops with varying maturity and yields, mix family and hired labour in different ratios and combine cultivation with other activities as needed to weather the effect of shocks (Chen 1991:111). Learning from India, Chen (1991) states that ‘engaging in multiple activities is an important way of promoting flexibility and encountering risk and uncertainty’ (Chen 1991:111). In India, when drought is predicted, shepherd households engage in agriculture. Other households engage in wage labour in order to accumulate resources which could help minimise the shocks (Chen 1991:111).

The mixing of crops has also been a strategy to prevent and reduce shocks from drought. In Tanzania and Kenya, mixing maize and cassava on the same plot can assist in surviving drought, as cassava is more resistant to dry conditions than maize (Mwaura 2008). Building granaries and storing food in times of abundance, to be used in times of scarcity, and building wells and dams have been effective in preventing and minimising the shocks. For instance, in Tanzania, the main vegetable for storage is the cowpea. When drought is predicted, the cowpea is cooked, dried in the sun and then stored. Other food stuffs ‘dried and stored included blood, meat and fish’ (Mwaura 2008:26). These are kept in traditional pots and baskets, which are hung above fireplaces (Mwaura 2008). Building up or drawing down inventories has also been highlighted as proactive household measures to ensure the availability of food. In normal years, households build up stocks that they can draw on during dry seasons. In Kenya, because of the unpredictable nature of drought, pastoralists embark on strategies to take advantage of the good years. Primarily, they often stock more productive females in their herds to ensure the cultivation of livestock, when the climatic conditions improve and grass and water become abundant, and thereby replace the animals lost to drought (Makali et al. 2010).

## Coping and responses

The concept ‘coping’ derives from observation of the manner in which people act using available existing resources and within a limited range of expectations to achieve various ends under stressful conditions (Davies 2000). Davies (2000:37) states that coping in general involves no more than managing resources; but, in this case, it is carried out in unusual, abnormal and adverse situations. Blaikie et al. (1994) argue that household strategies are often complex and involve a number of mechanisms for maintaining resources and livelihood in times of adversity.

In support of the above argument, Rahmato (1991) argues that indigenous disaster survival strategies involve the adoption of emergency resources management measures, the effective use of natural resources, divestment of savings and disposal of assets and the greater and more efficient use of the market system. Literature shows that coping strategies are confined to a number of areas. When a disaster strikes, households respond by ‘tightening their belts’ and consuming less food, drawing upon inventories which they have stored for contingencies when needed. If necessary, they dispose of non-productive assets, such as utensils, jewellery or other household items (Chen 1991:101). When no other options remain, they dispose of key productive assets, preferring mortgage to outright sale (Chen 1991). Under conditions of extreme shortage, some households are forced to take ‘drastic measures, such as migrating in search of food or abandoning a spouse or children’ (Chen 1991:120). As noted by Rahmato (1991):

[*T*]he sale or mortgage of assets or recourse to migration becomes operative at a later stage when other devices have by and large already been exhausted and should be regarded as true indicators of distress in a given scarcity period. (p. 137)

Examining farmers’ responses to drought in Southern Africa, Anderson et al. (2007) note that:

[*T*]ranshumance, or the seasonal migration of livestock, has long been recognized as an effective means of evading unfavorable climatic effects, such as drought, whereby moving domestic livestock across a landscape allows maximum forage use across a variety of climatic regimes and events. (p. 36)

Transhumance migration has long been proven to be effective not only in Southern Africa but also in other parts of the world, such as Ethiopia, Kenya, Tanzania, India, Bangladesh and the Sahel (Anderson et al. 2007; Rahmato 1991). Anderson et al. (2007) demonstrate that drought migration requires the movement of livestock and often entire families. Whilst migration has proven to be effective in weathering the effect of drought, it requires considerable effort and adjustment. As Anderson et al. (2007) show, in order to move:

[*F*]armers need networks and social linkages that extend into other ecological zones not created by drought. These networks need to be strong, and are often affected through family connections, as securing tenure in distant places can be controversial. (p. 38)

In this context, transhumance remains a challenge in South Africa, where land ownership remains a contentious issue.

In Kenya, pastoralists were successfully able to cope with droughts. One method adopted was to maintain exchange relations with neighbouring agricultural counterparts with whom they exchange livestock and animal products for grains to supplement their diets when the production of milk went down. The Maasai, for example, traded with neighbouring agricultural groups, such as the Kikuyu from Central Kenya, where they obtained cereals in exchange for their livestock products, such as milk, hides and skins (Orindi, Nyong & Herrero 2008). Under extreme cases, the Maasai have adopted non-pastoral activities like charcoal-burning or engage in various forms of employment for income. In some cases, raiding of neighbouring communities was also carried out for restocking (Orindi, Nyong & Herrero 2008). In addition, many households or families keep animals with relatives and friends elsewhere to guard against losses during drought. Animals kept elsewhere are always brought after the disaster, as pastoral families are able to restock quickly (Makali et al. 2009).

During times of drought, as well as famine that occur as a result of drought, parents who have family in unaffected areas send their children to their relatives to be cared for (Rahmato 1991:167). Peasants may travel long distances to remote areas unaffected by the drought to borrow food from a relative, a friend or someone with whom such arrangements can be made (Rahmato 1999:167). Brewing beer is also an important source of income, especially for rural women, as a reduction in grain ingredients caused by drought can affect their income and nutrition.

Oelofse and Dodson (1997), while examining poor rural households of the northern province of KwaZulu-Natal and the Eastern Cape, where restructuring of old age pensions has led to some pensioners being removed from the pension roll, found different coping mechanisms that have been employed as a result of this sudden shock. Households that had family networks, such as working children, and households which had savings and small businesses and were not fully reliant on pension money, were less vulnerable than households that had no family ties or reserves. The latter group was forced into a series of coping mechanisms such as borrowing or cutting back on food intake or taking children out of school. These actions made the households even more vulnerable.

## Recovery from drought

Are households able to return to their previous state of development after the drought has passed? The answer to this question is provided by Moser (1998) in his resilience and sensitivity approach. Moser (1998) believes that the nature of assets and risk management strategies are critical if one needs to understand how individuals or poor households recover from disaster. Here ‘asset’ refers to sources of income, such as employment and social capital, which include the social networks, skills and institutions responsible for disaster management. Moser (1998) reaffirms that the level of vulnerability to disaster, and the ability to recover from it, is determined by the quality and quantity of assets owned by an individual or a household.

Chambers (1983), using a case study in India, showed that, following the drought years, a decline in assets led to a circle of poverty, or what he calls ‘asset depletion-replenishment cycles’ where it is believed that those best able to replenish are those least depleted. This argument demonstrates how the lack of buffers to resist shocks can lead to other ‘ratchet’ effects that could result in a household becoming permanently vulnerable to poverty. Oelofse and Dodson (1997) contend that individuals or households that are highly vulnerable to injury, death and loss of property and disruption of their livelihood patterns are usually those who find it difficult to recover from disaster. Davies (2000) argues that the degree of resilience and sensitivity is important in explaining how households or individuals respond to changes in order to return to their previous state.

## Methodology

The methodology adopted for this study is a qualitative interpretive approach using mixed data collection methods. The key focus of qualitative interpretive research are subjective perceptions and understandings, which come from experience, objective actions or behaviours, and the context in which all of this occurs (Ulin et al. 2002).

For the purposes of this study, semi-structured interviews were conducted amongst 16 heads of household who were recruited from various areas of the Msinga villages, and participants in the study were over 19 years of age. The sample was heterogeneous as it was composed of both men and women. The objective of using male and female heads of household in the interview was to cross-check the information and see variations in view of both genders. Here, a household is understood as a unit, composed of the members of a family who live together under the same roof, whilst the head of a household is understood as a person in charge of all managerial activities and responsible for household needs.

In the semi-structured interview, questions are normally specified, but the interviewer was free to probe beyond the answers to seek clarification and elaboration. The selection of heads of households was justified by the fact that the researcher assumed that heads of a household would be better equipped to respond to the questions presented by the interviewer as they deal with all the households’ managerial decisions and the allocation of food during periods of drought. It was therefore expected that they would be informed about drought in the area. Sixteen respondents were chosen as they are placed in key positions that allowed them to interpret community vulnerability to drought and who understood how drought is managed. Busha and Harter (1980:56) argue that in the process of sampling, the ‘population can be very large or very small, depending upon the size of the group of persons or objects about which the researcher plans to make inferences’. In this study, the researcher was aware that the target population could only speak *isiZulu*. It was planned that, in the field work phase, the Zulu-speaking research assistants would do instant translation from English to *isiZulu* and vice versa to record the responses and make use of recorders to capture what might have been omitted whilst translating. Before the interview process, the research assistants underwent a three-hour briefing session on how to conduct interviews and provide instant translations.

In addition, the study used secondary sources of information and data. Secondary sources included books, journals and newspaper reports, obtainable through a library and online research. The secondary sources provided both quantitative and qualitative data as well as the theoretical framework. Secondary sources covered critical issues that are necessary to understand drought: its causes; relationship to vulnerability and poverty; and its socio-economic and political impact. It also helped explore the history of drought in South Africa and the policy implications.

### Ethical considerations

This article followed all ethical standards for research without direct contact with human or animal subjects.

## Findings

As discussed earlier, this study looks at the traditional knowledge approach that Msinga communities used in maintaining their livelihood in the face of climate change with a particular focus on drought. Particular focus is given to the drought preparedness and early warning, drought mitigation and prevention, coping and responses and finally, recovery from drought.

### Drought preparedness and early warning

The evidences emanating from this study demonstrated that communities in Msinga have vast knowledge on drought prediction and early warning. Most dominant are the observation of animal behaviour, ancestors’ revelations, migration of birds, and movement of winds and vegetation. Each point is presented below.

#### Behaviour of animals can indicate drought occurrence

During group discussions, the majority of participants agreed that the occurrence of drought can be determined by the behaviour of animals. Participants indicated that there are many signs from animals that can determine that drought is about to occur. The following quotes reads:

Cattle will lose weight as grass is dying. There will be foreign animals migrating to the areas and some indigenous animals will migrate elsewhere. You will always see goats and cows walking around houses and sometimes coming inside the houses and you will see some becoming very weak and can’t go far to look for grass. When we observe all these signs, then we will know that drought is close to us. (Participant 1, male, head of the household, age 40)

The behaviour of animals such as wild dogs and the cries of birds can determine whether there will be a rainy or dry year. Discussing how wild dogs can indicate the occurrence of drought, the participants argued that when there are many wild dogs in the area, and when they come around the houses, then it is probable that this particular year will face a severe drought. There is a common belief that wild dogs do not just come around the houses without a reason. The belief is that when wild dogs come around the houses it means the food in the wild has been exhausted:

It is rare to see a wild dog around the houses searching for food. When you see a wild dog coming around the houses looking for food, then know that will be a bad year. You will hear them crying around the houses and fighting with domestic dogs for food. (Participant 4, male, head of the household, age 42)

#### Ancestors can reveal when drought will occur

The role of ancestors is recognised in all aspects of life of rural men and women in the study area. It is commonly believed that nothing can happen to the community or individual without the ancestors’ consent and intervention. The role of ancestors in predicting drought is crucial in these communities. During group discussions, participants agreed that when drought is about to occur, ancestors will tell them through Izinyanga (diviners) or through an elder person. They indicated that the person who receives the message must inform members of the community so they can prepare for disaster:

Ancestors will always tell when anything bad is about to happen. They care for us. Sometimes they tell people what needs to be done in order to avoid such things from happening. If people do respect and honor what ancestors tell them to do, then nothing will happen, and if it happens, it cannot be very serious as it should. (Participant 4, male, head of household, age 42)

#### Vegetation can indicate when drought will occur

Another early drought occurrence indicator is the vegetation. All participants in the group discussions indicated that there are many signs from the vegetation that can demonstrate whether there will be a rainy or dry season. Amongst the indications discussed by participants are that, in June or July, there are indigenous trees which produce leaves and flowers. It is commonly believed that when these trees do not produce leaves and flowers at the expected time, then people will be aware that in such a year there will be severe drought:

Trees and grass, can tell you that this year, there will be rain or drought. When drought is about to happen, you will see trees, not growing any leaves, and will see that that the tree looks as dry. These trees normally bear leaves and flowers in June or July, and in August or later September, we have the rain. When this does not happen, people start getting ready for [a] dry year. (Participant 12, female, head of household, age 39)

#### Migration of birds is an indication of drought occurrence

The migration of birds can determine whether there will be drought or rain. According to the participants, in a year when drought is expected, some indigenous birds will migrate to other areas, whilst foreign birds migrate into the area. The migration of birds is not the only indicator of drought, but the cries of birds can also indicate drought occurrence:

Usually, we know that there will be drought or rain from observing and listening to birds. Sometimes, you will see all the birds that we usually see here, are no longer in the area. You will see other birds which are not of this area. Sometimes, birds usually sing when the rain is about to come, when we observe that these birds have not sung up to August, then [we] will expect serious drought in that year. (Participant 9, female, head of household, age 41)

#### Movement of winds indicate drought occurrence

The movement of wind has also been noted by participants as an early warning indicator of drought. During group discussions, the researcher asked how the movement of winds can determine drought occurrence. Participants in this category argued that:

When drought conditions are about to happen, you will always see the wind changing direction. Sometimes it is windy for a long period of time. For example it can be windy for three or six days successively, and when that happens, then we will know that drought is about to occur. (Participant 1, male, head of household, age 40)

### Drought mitigation and prevention

Numerous studies demonstrate that individuals or households devise a number of strategies to prevent their livelihoods from being affected by external shocks. The same literature indicates that individuals or households engage in multiple activities and promote flexibility to counter risk and uncertainty (Chen 1991; Mwaura 2008). Here, preparedness or prevention strategies are measures undertaken in order to prevent disaster from happening or, in case it happens, its impact can be minimised.

Examining households’ drought prevention strategies in Msinga, it can be concluded that there are four major drought preventative strategies:

1. praying to ancestors
2. growing drought-resistant crops
3. migration of men and young women to urban areas in search for work
4. grazing arrangements with distant communities.

Whilst analysing the effectiveness of these strategies, they presented some limitations in preventing drought from affecting livelihoods.

#### Praying to ancestors can prevent drought

The role of ancestors in preventing drought in Msinga is performed through rituals which connect the living and the dead, and seek protection from ancestors. It was argued by the participants that when drought is predicted, a number of ritual ceremonies are held to seek protection from ancestors. However, during group discussions, some participants, especially elders, were concerned about the behaviour of some members of the community abandoning their culture and adopting what they considered to be foreign ways and attitudes. They believed that the lack of respect and adherence to culture angered ancestors to the point where they allowed anything to happen in the community. The following quote reads:

When Izinyanga^1^ or other signs show that there will be a severe drought in a year, we have to go to the mountain and pray to our ancestors so they can protect us from drought. Drought [is] happening because some people in the villages are not paying tribute to our ancestors and sometimes do wrong things. When our ancestors get angry, they send us things like drought. We go up to the mountain early in the morning or afternoon and people have to stay there and pray to our ancestors, until the rain comes or our ancestors tell us to go back home. (Participant 4, male, head of household, age 42)

#### Growing drought-resistant crops can prevent drought

Growing drought-resistant crops and plants is another common prevention strategy that was noted throughout all the group discussions. This strategy is shared by both women and men. There are numerous indigenous crops and roots that have been introduced into agriculture systems in Msinga. These crops and plants are believed to be more resistant to drought. These crops include sorghum, a variety of maize, sweet potatoes and water melon. During group discussions, participants indicated that some of these crops have been introduced in later years, whilst others have been there for a long time:

Every year we are experiencing crop failure as [a] result of the lack of rain. Some species of crops which used to be grown are no longer grown as they cannot resist … the dry conditions. As you can see, there is no crop that can be grown in a very dry place like this. If we should have water, it could have helped, but we do not have water to replace the rain that we do not have, if the government could bring us some water, that would help a lot, as we can grow crops even when there is no rain. The only crops that can resist … dry conditions are sorghum and sweet potatoes, but people here do not like potatoes, you cannot eat potatoes every day. (Participant 6, male, head of household, age 38)

Despite drought-resistant crops and roots playing an important role during drought, they present some limitations. One of the limitations is that the production is low. It is also difficult to store them for a long period. For instance, participants indicated that sweet potatoes cannot be kept for a long time. They require immediate consumption after harvest. The researchers, examining the effectiveness of drought-resistant crops in protecting communities from starvation, noted that all participants argued that it brings very little relief and does not have much impact. This is evidenced in a participant’s statement:

Yes, we grow sweet potatoes, sorghum, but the problem is that, people cannot live on eating sweet potatoes for two or three months or even five months till the rain comes or not. If we have sweet potatoes, will it be possible to eat only potatoes without anything else? Sometimes you can’t even get these potatoes. (Participant 9, female, head of the household, age 41)

#### Migration can prevent drought from affecting livelihoods

The migration of men and young girls to urban areas in search of work is another strategy adopted by a number of households. Analysing the information from group discussions, it was apparent that this strategy is not shared by all members of the community. It may be that such a strategy is adopted by particular households. Migration to urban areas can be understood as one response to face the wider issue of poverty prevailing in Msinga. The level of unemployment, lack of basic services and lack of external support might have an influence on the migration of young men and women. Most of the male migrants work in mines, mainly in Johannesburg, whilst women do domestic work in towns, such as in Durban and its surroundings. Migration from Msinga as elsewhere in South Africa has a long history. It can be traced back to the discovery of mines during colonialism. Both domestic workers and mine workers have established strong social networks in cities. These networks help newcomers to find work by providing information and shelters when necessary (Participant 10, female, community member who works in urban areas, age 33).

#### Grazing land arrangements can ease the effect of drought

Grazing land with a distant community can ease the effect of drought especially to those who have cattle. However, this strategy is specific to men. Most women who participated in the group discussions were not aware of how grazing arrangements can prevent drought. Men indicated that there are grazing arrangements between traditional authorities in different traditional areas. When one area is affected, people can move and graze in the area which is not affected. Grazing arrangements presented some serious challenges. These challenges range from conflicts between those in search of grazing pastures and those living in the area. Another challenge is the distance that needs to be travelled and duration of stay whilst grazing. It was indicated by participants that those who have livestock also have other household responsibilities, and when they are absent from home, it creates more problems for the family left behind. Women asserted that when men are absent from home, it is difficult for women to negotiate loans and deal with other household problems. Taking care of the children whilst the man is away is a challenge to most of the women in this study. The absence of men adds more responsibilities to women who are already burdened with great responsibilities in caring for the household. The following quotes demonstrate challenges related to grazing with distant communities:

Grazing arrangements with other clans where there is no drought, helps keep our livestock alive, but the problem is that most of us do not have cows, only goats. It is difficult to travel two days or one week with goats in search of grass, if you are there what can you eat? You need to have enough food, now where can you get food to eat alone while you leave the family without food? (Participant 5, female, head of household, age 37)

We have problems going grazing in other areas. Sometimes you will find that people in the area are not happy with our presence, because they feel that we will exhaust all available grass. Sometimes this leads to conflict between those coming in the area and the owners of the land. (Participant 4, male, head of household, age 42)

The majority of participants agreed that prevention strategies do little to prevent drought:

Yes, people migrate with the cattle to other areas, people grow some crops, but all these contribute very little to prevent people from starving. Even those people who go somewhere else with their cattle, they do not return with all of them, some die. I know one neighbour who went [to] Nqutu, all his cows died, he only retuned with skins for drums. People these days prefer not to go, they just stay and wait. (Participant 2, male, head of household, age 42)

When the researcher asked what the participants think could be done to prevent drought from affecting their livelihoods, all participants stated the importance of water. They believed that if water is available to all households, it can be used for irrigating and livestock. The following statement was recorded:

Drought is not like a fire, no one knows where it comes from. If it was like a fire, everyone in the village would stand and we can put the fire down. But drought is something different. Maybe, if we should have water to irrigate like what I see in Dundee where there are white men, I think, we can reduce the impact of drought. Even those white people are affected by drought, even they that have those irrigation systems, but sometimes they grow crops and vegetables while we are suffering from drought and cannot get anything to eat. Only God and our ancestors can help us bring down this disaster. (Participant 7, female, head of household, age 45)

### Coping and responses

In the face of risks and uncertainties, households often develop several mechanisms for dealing with shocks and stress. Whilst examining households’ drought coping methods in Msinga, several strategies were identified. One of the strategies, which continues to dominate, is the prayer to ancestors; other ways include cutting down food intake, diversifying income, borrowing and dependence on relatives.

#### Pray to ancestors can mitigate drought effects

The participants consider that the role of ancestors is crucial in prediction, prevention and mitigating drought. During the group discussions, the participants indicated that praying to ancestors can mitigate the effect of drought. It was argued that when droughts are severe, old women organise themselves and go to seek protection from ancestors on the mountains. Researchers probed how such prayer is performed. One female participant argued that:

We choose some old women to go pray to God and ask for rain. When we come closer to the mountain, there is one tree from which we take some of leaves and wrap them on our hips and neck, then we go up to pray to God. Then God will tell us to go down to the river, and wash ourselves. When we arrive there, just after washing, the rain starts with us. Ah … ah … But these days people are not interested in these things, but sometimes we do, but not much as used to be … in that time. (Participant 10, female, head of household, age 43)

#### Cutting down food intake reduces the effects of drought

Cutting down food intake is another major coping strategy adopted by a number of households in Msinga. All focus groups indicated that they have gone one or more days without eating because of a lack of food. Some participants indicated that, during a good season, they eat two meals per day; and when there is drought, they eat one meal per day. In some instances, they do not get a full meal. Cutting down on food intake presented a number of problems. Some participants complained of getting ill and becoming weak due to insufficient food intake.

Cutting down food intake has gender implications. For instance, female participants argued that when there is a food shortage, the available food is allocated to children and husbands. Food allocation is the responsibility of women, and during food shortage as a result of drought, this causes problems for women’s household management strategies:

When there is a shortage of food, you do not know to whom to give food and whom to leave. First you think of the children, and then you have to think of their father as well. You cannot really know what to do. (Participant 12, female, head of household, age 39)

#### Diversifying income reduces the impact of drought

During times of uncertainty, communities in the study area engage in a number of activities. Evidences from group discussions revealed that there are very few opportunities in the areas, however, during drought, communities engage in small businesses, such as selling fat-cakes and other small items. During group discussions, it emerged that many participants were engaged in the business of selling basic items, such as sugar, soaps, chips and sweet. These have been identified by all participants as an alternative to escape the effects of drought. However, this strategy presents many weaknesses and limitations. One is that most of the households have no source of income to start such small business. For instance, one old woman claimed that she has never received more than R100 in the last 10 years. Another problem is that there are no formal markets where these items can be sold. Those engaged in this form of diversification have to market their product door-to-door, and in some instances sell to passers-by. This proved to be a difficult exercise for many of the participants.

Brewing traditional beer is another diversification strategy. Despite maize and sorghum becoming rare during drought, some women manage to get some maize and sorghum from distant communities. Beer is made and sold, helping women supplement the household’s income. Brewing beer has some challenges: when there is drought, people prefer to spend the money to buy food rather than beer. This affects the beer market as there is no money. In some instances, when people run out of money to buy traditional beer, in this instance, it is sold in exchange for a service, such as helping the brewer cultivate or any other services he or she may require. Some of participants argued that due to these challenges linked to the diversification of income they end up staying at home and waiting until the drought is over. In one instance, an old woman indicated that when there was a severe drought in 2004, she bought some food items, but due to storage problems, the items were destroyed. There were other households that were engaged in selling meat and food along the way to travellers. They said:

When we have nothing else to fall back on, we just do whatever gives us some money, and then we go to buy food from Pomoroy. When there is no rain, everyone get[s] involved in business. But [it] is very difficult, people just try. (Participant 1, male, head of household, age 40)

#### Borrowing can reduce the impact of drought

Borrowing money to reduce the effects of drought is another strategy adopted by some households in the study area. Men are more involved in borrowing money, whilst women borrow food from relatives or friends. Men are believed to be better than women in negotiating money. However, women are also good in negotiating for food and other household items, such as salt or soap from neighbours or friends. Amongst women, there is a spirit of sharing. When one woman has no items, such as salt or soap, she can go to her neighbour and get these items. For men, during group discussions, participants said that when drought occurs and when there are no other options, borrowing money becomes one of the options. There are no formal lending agencies such as banks, but communities have established informal lending structures called *Mashonisani.* Borrowing systems present a number of problems. One is that when a borrower is not able to pay back the money, he or she may be required to work for the lender in return. However, participants complained about some lenders who take advantage of the vulnerable situation of the communities by hiking interest rates. Other participants complained about the behaviour of some lenders when it comes to demanding their money back:

[*H*]ere we are poor and we do not have money to meet our needs, for those of us who do not have money, when we get money from them and you do not have the means to pay back, then they come hard on you and they will demand their money by force, now things are changing, it was not like this before, during our times people were very supportive. (Participant 3, male, head of household, age 42)

#### Social support can reduce drought effects

Support from relatives has a role to play for many women who participated in the discussions. During focus group discussions, it was evident that when women have relatives, such as working husbands in urban areas and other relatives, they send money in the form of remittances. Support from relatives presented some limitations. One is that the money is not received when it is most needed. Participants, who have working relatives in cities, argued that money is received once a month or after two months, due to transaction problems or the lack of availability of money. They said that working relatives sometimes do not get money to send home as a result of low wages and increases in the cost of living in urban areas. The following statement demonstrates challenges related to the relatives dependence:

I have no other source of income. I only rely on my daughter who is working in Durban as a domestic worker. Sometimes, she doesn’t get the money on time, because her boss did not pay her. If it happens, then we have to struggle and find other ways. Sometimes we borrow food stuff[s] from a local shop dealer, when my daughter send[s] us the money, then we pay the shop dealer. (Participant 7, female, head of household, age 45)

### Recovery from drought

Having looked at coping strategies during and after the drought, it is evident that household systems are not stable but change over time as they respond to a particular crisis and are therefore not equally vulnerable to the same risk (Davies 1996; De Satge et al. 2002; Oelofse & Dodson 1997). A greater percentage of participants lack the capacity to withstand shocks. They are unable to recover from drought given their income levels, and the lack of social and external support. They could thus be regarded as a ‘low resilient household’ since they have been unable to resist the effects of the disaster. These low resilient households are faced with the after-effects of drought that restrain them from recovery. In Davies’ (1996) words, ‘their capacity to recover has been eroded’, and they cannot even meet their basic needs. Their main concern is survival, and they will therefore make every effort to earn a living rather than being too worried about the future. In this study, very few participants displayed a capacity to recover or gave an indication that they will recover in the future. For example, one man indicated that it has taken him more than 10 years to buy a cow, thus implying that it might take him more or less the same number of years to recover.

## Conclusion

This study looks at the traditional knowledge Msinga communities use in maintaining their livelihood in the face of extreme climatic conditions with a particular focus to drought.

This article covered the review of the literature on the subject matter and discussed the results of the study. The review of the literature on this subject showed that traditional knowledge is still popular in some communities, especially, those not influenced by modern forms of knowledge, and are intertwined in their ways of life. It is difficult for its proponents to prove to the scientific world the power such knowledge holds in dealing with many aspects of life. As this review has shown, local or traditional knowledge has strengths and weaknesses.

However, for the common goal of achieving the well-being for all, it is desirable that respective strength is built upon through co-operation and mutual exchange of experiences. Positive attempts of collaboration have been reported from some developing countries. This review has shown that an effective, user-oriented, community-based partnership between the modern and traditional knowledge holders should be implemented. However, there has been relatively little experience in this regard.

The finding from interviews revealed that communities in the study area have traditional knowledge to predict, prevent, mitigate and cope with drought, such as cultivation of drought-resistant crops, seasonal migration and diversification of the market. However, this knowledge presents many weakness and limitations. One is that since the level of poverty is high, communities do not have assets to convert into other consumable assets. The community’s traditional knowledge is dying out, which is vaguely understood by all members of the community. Communities have no means to diversify their income when drought is severe. Combining all these factors, there is a reason to believe that they are vulnerable and unable to resist the drought effects.

## Footnotes

1 In IsiZulu or the Zulu language, ‘Izinyanga’ are the experts who predict unexpected events, such as natural catastrophes (i.e. droughts, floods, diseases), and who can also predict good or bad season for a particular period. In the case of death and other misfortune happening in ones’ life, Izinyanga are consulted to determine the causes.
